# Application of artificial neural network model in diagnosis of Alzheimer’s disease

**DOI:** 10.1186/s12883-019-1377-4

**Published:** 2019-07-08

**Authors:** Naibo Wang, Jinghua Chen, Hui Xiao, Lei Wu, Han Jiang, Yueping Zhou

**Affiliations:** 1 Jiangxi Province Key Laboratory of Preventive Medicine, Nanchang University, Nanchang, 330006 People’s Republic of China; 2 Jiangxi Centre for Health Education and Promotion, Nanchang, China; 3 Second Affiliated Hospital, Nanchang University, Nanchang, China

**Keywords:** Alzheimer’s disease, Artificial neural network, Urban communities, Risk factors, Early diagnosis

## Abstract

**Background:**

Alzheimer’s disease has become a public health crisis globally due to its increasing incidence. The purpose of this study was to establish an early warning model using artificial neural network (ANN) for early diagnosis of AD and to explore early sensitive markers for AD.

**Methods:**

A population based nested case-control study design was used. 89 new AD cases with good compliance who were willing to provide urine and blood specimen were selected from the cohort of 2482 community-dwelling elderly aged 60 years and over from 2013 to 2016. For each case, two controls living nearby were identified. Biomarkers for AD in urine and blood, neuropsychological functions and epidemiological parameters were included to analyze potential risk factors of AD. Compared with logistic regression, k-Nearest Neighbor (kNN) and support vector machine (SVM) model, back-propagation neural network of three-layer topology structures was applied to develop the early warning model. The performance of all models were measured by sensitivity, specificity, accuracy, positive prognostic value (PPV), negative prognostic value (NPV), the area under curve (AUC), and were validated using bootstrap resampling.

**Results:**

The average age of AD group was about 5 years older than the non-AD controls (*P* < 0.001). Patients with AD included a significantly larger proportion of subjects with family history of dementia, compared with non-AD group. After adjusting for age and gender, the concentrations of urinary AD7c-NTP and aluminum in blood were significantly higher in AD group than non-AD group (2.01 ± 1.06 vs 1.03 ± 0.43, 1.74 ± 0.62 vs 1.24 ± 0.41 respectively), but the concentration of Selenium in AD group (2.26 ± 0.59) was significantly lower than that in non-AD group (2.61 ± 1.07). All the models were established using 18 variables that were significantly different between AD patients and controls as independent variables. The ANN model outperformed the other classifiers. The AUC for this ANN was 0.897 and the model obtained the accuracy of 92.13%, the sensitivity of 87.28% and the specificity of 94.74% on the average.

**Conclusions:**

Increased risk of AD may be associated with higher age among senior citizens in urban communities. Urinary AD7c-NTP is clinically valuable for the early diagnosis. The established ANN model obtained a high accuracy and diagnostic efficiency, which could be a low-cost practicable tool for the screening and diagnosis of AD for citizens.

## Background

With the enlarging proportion of aging population, the incidence of Alzheimer’s disease (AD) has been increasing, which no doubt becomes a public health crisis globally [1]. AD will seriously affects the quality of life of patients, and there are no ideal drugs or methods for clinical treatment [2]. *2016 Alzheimer’s disease Facts and Figures* has reported that AD led to 84,767 deaths recorded by official death certificates in 2013 and became the fifth leading cause of death in elderly Americans over 65. In 2015, the time of care provided to patients with AD and other dementias was nearly 18.1 billion hours which valued more than $221 billion [1]. The data above shows that substantial burdens have been placed on the family, society and state. In china, Alzheimer’s disease is becoming the fastest growing fatal disease and at least 9.5 million patients have been diagnosed by far. Nearly 1 million new cases will be found every year and the number of new cases is increasing year by year.

Although researchers have revealed a great deal concerning AD, much is yet to be discovered about the precise pathogenesis, owing to its complex causes involving genetic, environmental and metabolic factors. Wide ranges of explorations in view of social medicine, epidemiology and molecular medicine have been carried out and most hold the view that AD is caused by multidimensional factors related to physiology, psychology and sociology [3–10]. The ways of screening for patients with AD relies heavily on clinical manifestations and neuropsychological scales including Mini-mental State Examination (MMSE), Montreal Cognitive Assessment (MoCA), Activities of Daily Living (ADL) and Global Decline Scale (GDS). These approaches are practicable for patients with advanced AD, whereas early detection will be key to preventing, slowing and stopping Alzheimer’s disease. Therefore, experts and scholars around the world have made active exploration in predicting AD, concentrating on risk factors and biomarkers. The accumulation of the protein beta-amyloid outside neurons and an abnormal form of the protein tau inside neurons are two of several changes believed to contribute to the development of AD. Biomarkers believed to be useful for detection for AD include amyloid beta (Aβ), T-tau, P-tau, ApoE-ε4 and Alzheimer-associated neuronal thread protein (AD7c-NTP) [11–14].

The main existing methods of AD diagnosis are magnetic resonance imaging (MRI) of brain, genetic detections of some proteins or other pathological detections. These methods that are mainly for clinical patients cost much time and money, and they are especially not suitable for early screening and diagnosis of large population. With the deepening of etiological research of AD, studies of multi-dimensional factors concerning environment, inheritance and life behavior have been carried out. Many experts have taken advantage of the results of etiology to establish different mathematical models [15–17], which deeply promote to the research of early diagnosis and prediction of AD. Because of the complexity of AD, the relationship between various risk factors is not independent, resulting in the situation that most traditional statistical models are not very applicable. However, artificial neural network has a nonlinear adaptive processing system made of a large number of artificial neurons in the right way to connect each other [18]. With the ability to approximate any function with arbitrary precision, this model can solve the problems of uncertain or ambiguous medical information more effectively. In our previous study, an ANN containing information of trace elements and neurotransmitters had been established and could accurately distinguish between AD patients and non-AD controls [19]. In this study, epidemiological parameters, scales of neuropsychological functions and biomarkers were combined together, and the back-propagation neural network of three-layer topology structures was applied to develop an early warning model in order to provide effective method for early diagnosis of AD and to explore early sensitive markers for AD.

## Methods

### Study subjects

The cohort of our project was established in Hongdu community with a large and stable population in Nanchang, China in 2012. Senior citizens aged 60 years and over in this community were recruited and a follow-up cohort of 2482 residents had been formed by the end of 2012. Nested case-control study design applied in this study, 89 new AD cases with good compliance who were willing to provide urine and blood specimen were selected from the cohort from 2013 to 2016. Every two controls were picked when discovering a new case. The criteria of choosing for controls was the nearest residential address. The control living in the closest floor had been chosen if suitable controls lived in the same building. AD diagnosis was performed by experienced neurologists according to ICD-10 and National Institute of Neurological and Communicative Diseases and Stroke—Alzheimer’s disease and Related Disorders Association criteria [20].

### Survey

A standardized questionnaire was developed for collecting data on 19 items in four dimensions: demographic characteristics (age, gender, education, previous occupation, marital status, monthly family income), behavioural characteristics (tobacco smoking, alcohol drinking, physical exercises, social activities, living alone or not, personality), medical history (diabetes, hypertension, Parkinson, traumatic injury of brain, family history of dementia) and neuropsychological performance assessments (MMSE and ADL). The MMSE scale can comprehensively and accurately reflect the intelligence status and cognitive impairment of the subjects. The ADL scale can quickly assess the ability of daily living activities of the subjects. Both these two scales contribute to the screening of AD. The definitions of behavioural characteristics were as follows: 1) Smoking was defined as one or more cigarettes per day and the duration was more than 1 year before the investigation. 2) Alcohol drinking was defined as drinking alcohol at least 2 times a week for more than 1 year before the investigation. 3) The Frequency of physical exercise and social activities was defined as: “regularly” means more than 3 times a week, “sometimes” means 1–2 times a week and “never” means less than once a week. In addition, the individuals with family history of dementia refer to the subjects of the first-degree relatives with dementia. The data were collected by strictly trained investigators and the information of medical history was obtained from health records stored in the Health Service Centre of community. The incomplete questionnaires were filled out by interviewing the subjects in their homes again.

### Laboratory assays

Five biomarkers comprised of trace elements (aluminum, selenium and zinc), urinary AD7c-NTP and Aβ42 in blood were assayed. The concentration of AD7c-NTP (urine) was tested in strict accordance with ELISA kit (Hanke, Hainan, China) and so does the concentration of Aβ42 (blood) with ELISA kit (Excell Biology, Shanghai, China). The detection of trace elements was carried out using atomic absorption spectroscopy (AAS) and graphite atomizer with reference standard provided from the state.

### Statistical analysis

Data were entered and analyzed using Epidata 3.1 and SPSS 21.0. Descriptive characteristics were presented as mean and SD for continuous variables or frequency and percentage for categorical variables. Comparisons between AD and non-AD groups were made with t-test for continuous variables and chi-square analysis for categorical variables. Generalized linear regression was used for the adjusting of age and gender when comparing the scores of MMSE and ADL, the concentrations of biomarkers in urine and blood between cases and controls. Level of statistical significance was set at a 2-tailed *p* < 0.05.

### ANN model

Due to the request of sigmoid transfer function, the original data should be normalized to range from 0 to 1, of which the purpose is to avoid big training error resulted by the difference of quantity level between input data and output data. All data were normalized to a range from 0 to 1 using the range method (${\mathrm{x}}_{\mathrm{i}}^{,}=\frac{{\mathrm{x}}_{\mathrm{i}}-{\mathrm{x}}_{\mathrm{min}}}{{\mathrm{x}}_{\mathrm{max}}-{\mathrm{x}}_{\mathrm{min}}}$).

After being converted appropriately, the data were randomly divided into training set (70% of the samples) and testing set (30% of the samples). This ratio is chosen from other two different ratio combinations used. The first combination was with 66.7% of the inputs for training and 33.3% of the inputs for testing, and the second combination is 75% (training) and 25% (testing). But the best performance of ANN was obtained with 70% of the data for training and the rest for testing.

Taking the complexity of the network, training time and “over-fitting” into account, the neural network designed in this study consists of three layers (one input layer, one hidden layer and one output layer). The input layer consists of 18 neurons (18 variables that were statistically significantly different between the cases and the controls) as network inputs. Each neuron performs a weighted summation of the inputs. The activation function was sigmoid function ($\mathrm{f}\left(\mathrm{x}\right)=\frac{1}{1+{\mathrm{e}}^{-\mathrm{x}}}\Big)$. Training algorithm for ANN was the most widely used Back propagation (BP) algorithm. It is generally believed that the BP-ANN network model needs 5–10 times the number of variables in input layer to ensure the reliability and external validity [21], and our sample size meets this demand.

The ANN model was established with SPSS statistics client and was evaluated using the diagnostic test including sensitivity, specificity, accuracy, positive prognostic value (PPV), negative prognostic value (NPV) and area under curve (AUC). Bootstraps with 1000 resample were used for validity of the ANN model.

### Logistic regression, k-nearest neighbor (kNN), support vector machine (SVM) model

For the purpose of testing the advantage of NN algorithm, logistic regression model, k-Nearest Neighbor (kNN) and support vector machine (SVM), were applied using the same 18 variables that were significantly different between AD patients and controls as independent variables to make a comparison with ANN.

For the logistic regression model, previous occupation, marital status and personality were set as dummy variables. 0.5 was used as a prediction threshold value. The proportion of training set and testing set was the same as ANN, and Bootstraps with 1000 resample were also used for the validity of efficacy. To evaluate and compare the predictive accuracy of these models, we also calculated sensitivity, specificity, accuracy, PPV, NPV and AUC.

R software version 3.5.2 (R Development Core Team, Vienna, Austria) was used for our analysis. The following R packages for machine learning approaches were used: caret, e1071,nnet.

## Results

### Demographic characteristics

Table 1 shows that the average age of AD group (77.44 ± 6.82) was about 5 years older than non-AD group (72.49 ± 6.86), and the proportion of females in cases (69.66%) was larger than that in controls (49.44%). Moreover, significant difference was found in education, previous occupation, marital status and monthly family income.

**Table 1:** Demographic characteristics of AD and non-AD groups

	AD(*n* = 89)	Non-AD (*n* = 178)	t/X^2^	P
Age (mean ± SD)	77.44 ± 6.82	72.49 ± 6.86	5.56	< 0.001
Gender [n(%)]				
male	27 (30.34)	90 (50.56)	9.86	0.002
female	62 (69.66)	88 (49.44)		
Education [n(%)]				
illiteracy	36 (40.45)	18 (10.11)	40.12	< 0.001
primary	24 (26.97)	43 (24.16)		
junior	27 (30.34)	99 (55.62)		
Previous occupation [n(%)]				
worker	55 (61.80)	125 (70.22)	32.17	< 0.001
farmer	27 (30.33)	19 (10.67)		
Administrative cadre	3 (3.37)	10 (5.62)		
technician	4 (4.50)	24 (13.48)		
Marital status [n(%)]				
married	53 (59.55)	129 (72.47)	14.22	0.001
unmarried	6 (6.74)	0 (0.00)		
divorced or widowed	30 (33.71)	49 (27.53)		
Monthly family income [n(%)]				
0-	36 (40.45)	0 (0.00)	83.75	< 0.001
1000-	16 (17.98)	49 (27.53)		
2000-	25 (28.09)	95 (53.37)		
3000-	12 (13.48)	34 (19.10)		

### Behavioural characteristics

There was no statistically significant difference in smoking and frequency of social activity between the cases and the controls. Non-AD group had significantly higher frequency of physical exercise and lower proportion of loneliness (11.80% vs 24.72%). Additionally, Alcohol drinking and type of personality were significantly different between the cases and the controls. More details could be found in Table 2.

**Table 2:** Behavioural characteristics of AD and non-AD groups n (%)

		AD (*n* = 89)	Non-AD (*n* = 178)	X^2^	p
Smoking	yes	26 (29.21)	43 (24.16)	0.792	0.374
	no	63 (70.79)	135 (75.84)		
Alcohol drinking	yes	29 (32.58)	86 (48.31)	5.99	0.014
	no	60 (67.42)	92 (51.69)		
Physical exercise	never	27 (30.34)	24 (13.48)	12.37	0.002
	sometimes	16 (17.98)	28 (15.73)		
	regularly	46 (51.69)	126 (70.79)		
Social activity	never	23 (25.84)	41 (23.03)	2.31	0.315
	sometimes	37 (41.57)	62 (34.83)		
	regularly	29 (32.58)	75 (42.13)		
Living alone	yes	22 (24.72)	21 (11.80)	7.33	0.007
	no	67 (75.28)	157 (88.20)		
Personality	introverted	33 (37.08)	53 (39.78)	7.25	0.027
	intermediate	23 (25.84)	29 (16.29)		
	extroverted	33 (37.08)	96 (53.93)		

### Medical history

AD group included a significantly larger proportion of subjects with history of diabetes (39.33% vs 26.97%), as well as Parkinson (8.99% vs 2.25%) and family history of dementia (17.98% vs 6.18%), compared with non-AD group. There was no statistical difference in the proportion of hypertension and traumatic injury of brain between the cases and the controls (Table 3).

**Table 3:** History of diseases of AD and non-AD groups n (%)

	AD (*n* = 89)		Non-AD (*n* = 89)		X^2^	p
	yes	no	yes	no		
Diabetes	36 (39.33)	54 (60.67)	48 (26.97)	130 (73.03)	4.09	0.043
Hypertension	39 (43.82)	50 (56.93)	82 (46.07)	96 (53.93)	0.12	0.728
Parkinson	8 (8.99)	81 (91.01)	4 (2.25)	174 (87.75)	6.28	0.013
traumatic injury of brain	5 (5.62)	84 (94.38)	18 (10.11)	160 (89.89)	1.52	0.217
Family history of dementia	16 (17.98)	73 (82.02)	11 (6.18)	167 (93.82)	9.09	0.003

### Neuropsychological functions

In cases, the score of MMSE was significantly lower (17.64 ± 5.38 vs 26.57 ± 3.63), and the score of ADL was significantly higher (31.73 ± 11.71 vs 15.26 ± 7.90) compared with the controls after adjusting for age and gender (Table 4).

**Table 4:** Comparisons of the scores of MMSE and ADL between cases and controls mean ± SD

	AD(n = 89)	Non-AD(*n* = 178)	t	p
MMSE	17.64 ± 5.38	26.57 ± 3.63	21.94	< 0.001
ADL	31.73 ± 11.71	15.26 ± 7.90	18.57	< 0.001

Note: age and gender were adjusted

### Biomarkers in urine and blood

The concentrations of urinary AD7c-NTP and aluminum in blood were significantly higher in AD group than non-AD group (2.01 ± 1.06 vs 1.03 ± 0.43, 1.74 ± 0.62 vs 1.24 ± 0.41 respectively). The concentration of Selenium in AD group (2.26 ± 0.59) was significantly lower than that in non-AD group (2.61 ± 1.07). However, there was no statistically significant difference in Aβ42 (blood) and Zinc between cases and controls. Age and gender were adjusted when comparing these variables (Table 5).

**Table 5:** Comparisons of concentration of biomarkers between the cases and the controls mean ± SD

	AD(n = 89)	Non-AD(n = 178)	t	p
AD7c-NTP (ng/ml)	2.01 ± 1.06	1.03 ± 0.43	116.86	< 0.001
Aβ42 (pg/ml)	38.49 ± 16.72	50.12 ± 21.31	−1.74	0.083
Aluminum (μmol/ml)	1.74 ± 0.62	1.24 ± 0.41	123.42	< 0.001
Selenium (μmol/ml)	2.26 ± 0.59	2.61 ± 1.07	−23.55	< 0.001
Zinc (μmol/ml)	61.24 ± 15.21	64.03 ± 7.92	−1.39	0.165

Note: age and gender were adjusted

### The comparison of ANN, logistic regression, kNN and SVM

Table 6 presents the efficacy of classification of AD in resampling testing sets of established four models. In the testing sets, the average sensitivity of ANN model was 87.28%, and the specificity was 94.74%. The accuracy of ANN was 92.13%, which was higher than that of logistic regression model. The area under curve (AUC) for these four models were 0.897, 0.804, 0.832 and 0.864 respectively.

**Table 6:** The efficacy of ANN, logistic regression, kNN and SVM in testing sets

Assessment index	ANN (95% CI)	Logistic regression (95% CI)	kNN (95% CI)	SVM (95% CI)
Sensitivity (%)	87.28 (85.12–88.34)	71.36 (69.18–72.93)	75.42 (73.24–76.17)	84.23(82.78–85.63)
Specificity (%)	94.74 (92.61–95.75)	90.25 (87.82–91.85)	89.39 (87.65–90.49)	90.45(88.65–91.73)
Accuracy (%)	92.13 (89.48–92.57)	83.92 (80.31–85.11)	84.91 (83.01–86.41)	86.04(84.93–88.10)
PPV (%)	88.64 (86.83–90.06)	73.11 (71.09–74.86)	83.02 (82.17–85.23)	82.36 (81.25–83.94)
NPV (%)	91.28 (90.41–93.10)	86.37 (85.19–88.26)	86.41 (84.85–88.15)	88.77(87.21–90.33)
AUC	0.897 (0.877–0.915)	0.804 (0.781–0.818)	0.832 (0.817–0.849)	0.864 (0.853–0.876)

## Discussion

The results in this study indicate that age, lower education level and monthly family income may increase a person’s risk for developing AD in urban communities in China. As is known to all, age is the greatest risk factor of AD. But it is not a normal part of aging and age alone is not sufficient to cause the disease. The function of brain decreased significantly with aging. The obvious manifestation of nervous system of aging is the cognitive decline, including memory, attention, learning ability and visual function [22, 23]. Knowledge, as a stimulus, can promote the growth of dendrites and axons in brain cells, improving the compensatory capacity of the aging of brain and reducing the degree of cognitive impairment. Therefore the incidence of AD differs in individuals of different educational levels [1]. Furthermore, educational levels may affects personal occupation and social status, which may also have an influence on the development of AD. In this study, monthly family income of non-AD group was significantly higher than AD group. Higher monthly family income means richer daily life and more medical resources, suggesting that it is one of protective factors for AD [24]^.^

Many studies have indicated the fact that cognitive decline is significantly associated with dietary habits and lifestyle involving physical exercise and reading [25–29]. It is worth noting that in our study, drinking subjects in non-AD group were significantly more than that in AD group, suggesting moderate alcohol drinking may be beneficial to prevent AD. Experts have certificated that alcohol consumption can reduce the risk of AD, which is related to the interaction of polyphenols and Tau protein [30]^.^ Regardless of alcohol consumption, only the frequency of drinking was recorded in our study, which may be the reason to this result. Current researches suggest diabetes can increase the viscosity of blood and lead to cognitive impairment [31]^.^ Insulin resistance may also increase the level of inflammatory factors and lower the utilization of blood glucose. That pathological mechanism could accelerate the accumulation of amyloid and toxic substances in brain, resulting in increased risk of AD [32]. In addition, Individuals who have a parent, brother or sister with AD are more likely to develop the disease than those who do not have a first-degree relative with AD [33, 34], which could be explained by the genetic factors more or less.

A certain degree of elevation of AD7c-NTP existing in brain tissue, cerebrospinal fluid and urine could be found in patients with AD of early and middle stages, and the content of AD7c-NTP is positively correlated with the severity of disease [35]. One study has demonstrated that the sensitivity and specificity of urinary AD7c-NTP can reach more than 90% when screening AD [36]. The increased AD7c-NTP in brain tissue can enter the blood through the blood-brain barrier, and eventually go into the urine by glomerular filtration. Urine specimen has the advantages of being non-invasive, relatively cheap and easily available, compared to brain tissue and cerebrospinal fluid, so urinary AD7c-NTP could be utilized as a potential and valuable molecular biomarker for the early diagnosis of AD. There was no difference between patients with AD and controls in terms of Aβ42 in blood in this study, indicating that its clinical value needs further discussion. The concentration of aluminium and selenium were found still significantly diverse in AD and non-AD groups as our previous study [23]^.^ However, zinc was found related to aging in our recent research but it seemed not associated with AD.

In view of the characteristics of AD, such as the slow onset, difficult treatments, and heavy disease and social burdens, it is critical to develop simple, economic, reliable and efficient methods for early discovery and diagnosis, which is the key of this study. There are several existing early diagnosis and prediction models for AD, but they may have some limitations. Hye [37] tried to use 10 kinds of plasma proteins associated with AD to predict this disease, but found the sensitivity and specificity of them were all lower than 90%. Some ANN models applied in diagnosis of AD were better than the model in this study. For example, Grossi [38] use the counts of neurofibrillary tangles and neurotic plaques in cerebral cortex and hippocampus as input variables to build an ANN model. Although it finally could perfectly distinguish Alzheimer’s patients from controls and the accuracy could reach 100%, the input variables in their model mostly include invasive clinical examinations or complex laboratory tests, which may be not practical for screening of large populations. Even if our team had established an ANN containing information of trace elements and neurotransmitters with a relatively high accuracy of 92.5% [19], it is not convenient to get all those biological data. All in all, in our established ANN model, the input variables consisting of demographic characteristics, behavioural characteristics, medical history, neuropsychological performance and biomarkers were rigorously selected and significantly associated with AD. Additionally, NN algorithm has shown its advantage in the efficacy of prediction in contrast with other classifiers. This ANN model is more comprehensive, economical and easily available, compared to clinical examination such as CT and MRI.

## Conclusions

Increased risk of AD may be associated with higher age. Lower education level and monthly family income, Family history of dementia and physical inactivity may all lead to the developing of AD. Urinary AD7c-NTP is clinically valuable for early diagnosis of AD, but Aβ42 in blood needs further discussion. The final established ANN containing multiple information including epidemiological parameters, neuropsychological functions and biomarkers obtained a high diagnostic precision and efficiency. It can be viewed as a low-cost practicable tool for the screening and diagnosis of AD.

## Data Availability

The data sets of the current study are available from the corresponding author on reasonable request.

## Abbreviations

AD: Alzheimer’s disease
AD7c-NTP: Alzheimer-associated neuronal thread protein
ADL: Activities of Daily Living
ANN: Artificial neural network
Aβ: Amyloid beta
BP: Back propagation
GDS: Global Decline Scale
MMSE: Mini-mental State Examination
MoCA: Montreal Cognitive Assessment
MRI: Magnetic resonance imaging
